# Swiss Hospitals and Their Administration

**Published:** 1912-07-20

**Authors:** Conrad W. Thies

**Affiliations:** formerly Secretary, Royal Free Hospital, London.


### SWISS HOSPITALS AND THEIR ADMINISTRATION.

### With Special Reference to the Cantonal Hospital of Geneva.

By CONRAD W. THIES, formerly Secretary, Royal Free Hospital, London.

In the first place, I would point out that there is no uniformity in the system of hospital administration throughout the Swiss Confederation. Each of the twenty-two cantons makes its own independent arrangements for the treatment of its sick and poor citizens. The Federal Government does not subsidise the hospitals, or exercise any control over this department of public work, which is entirely in the hands of the Councils of the various cantons. From inquiries made in different cantons, I learn that, notwithstanding this absence of any central authority which could enforce uniformity of methods, the work of providing accommodation for the reception and treatment of the sick is generally speaking carried out on somewhat similar lines throughout the Swiss Confederation. In these circumstances some particulars of the regulations and arrangements which are in force in Geneva will fairly describe the methods adopted by the other cantons.

The Cantonal Hospital of Geneva provides 700 beds, and receives each year about 7,000 in-patients. The buildings were originally erected in 1855, and have been added to and altered from time to time. At the present time an entirely new wing is in course of construction to accommodate the surgical wards, operating theatres, and x-ray installation, etc. In reference to the regulations for the admission of patients to the cantonal hospitals, it must be borne in mind that there are no absolutely free beds in any of the public hospitals of Switzerland, such, for instance, as are provided in the voluntary hospitals, Poor-Law infirmaries, and infectious diseases hospitals of Great Britain. Every patient who is admitted into a Swiss hospital must be paid for. Those persons who are destitute, or who are too poor to be able to pay anything towards the cost of their treatment, are paid for by the Board of Public Assistance. All other classes of patients must pay, either directly or through the Trade and Friendly Societies, of which they are members, the rate of payment being fixed by the hospital authorities after due consideration of the means and circumstances of the patient. All patients before admission must furnish full information as to their means and so forth on a printed form supplied by the hospital, and this form must be duly certified by a medical practitioner, or by the recognised local authority of the district where the patient resides. When necessary the particulars given are verified by local inquiries, and any person making a false declaration is liable to severe civil penalties.

The charges made to the various classes of patients are regulated according to their circumstances. In Geneva, for instance, working-class persons who have been resident in the canton for not less than six months are charged from 2 to 3 francs per day; Swiss citizens from other cantons pay 3.50 francs; while aliens, or foreigners, pay from 3.50 to 5 francs. In the case of poor Swiss from other cantons who cannot without risk to health be sent to their own district, the charges are defrayed by the Public Assistance Board, which takes steps to recover the amount, either from the patient or from the canton to which he belongs. When a poor patient, on recovery of health, finds remunerative employment, he is liable to repay at least a portion of the cost of his hospital treatment to the Board; or should such a patient die in hospital, leaving any property, the Board have a first claim upon his estate for the cost of his treatment and funeral expenses. Provision is made in all the cantonal hospitals for the reception of middle-class patients, who, however, can only be admitted upon a certificate signed by their medical practitioner, stating the nature of their illness and other particulars. This class of patients are charged from 7 to 15 francs per day, according to the kind of accommodation which they require. In maternity cases, or where an operation is needed, the patient has also to pay an extra charge for dressings, etc.

The income of the cantonal hospitals is derived from various public and private sources. From the Report of the Geneva Hospital for 1910 I find that the total income for that year was, in round figures, 955,000 francs (£38,200), made up as follows, viz.:

| | Francs | £ |
|---|---|---|
| Grants from the Cantonal Council of Geneva | 450,000 | (18,000) |
| Payments by the Board of Public Assistance for the treatment of poor patients | 350,000 | (14,000) |
| Payments by patients and their friends | 90,000 | (3,600) |
| Payments by Societies for their members | 10,000 | (400) |
| Annual collection made throughout the whole Canton | 25,000 | (1,000) |
| Donations and legacies | 20,000 | (800) |
| Sundry receipts | 10,000 | (400) |
| Total income | 955,000 | (£38,200) |

It should be added that the above amounts are exclusive of sums which have been received for such special purposes as new buildings, or from legacies which have been left in trust. It is interesting to learn that the property of all citizens and residents in the Canton of Geneva who die intestate or without legal heirs goes to the hospitals.

The cost of the land and the erection and equipment of the hospital has been met by special grants from the Cantonal Council, supplemented by donations and collections, much the larger portion of the cost being defrayed out of public funds. The new surgical wing now in course of erection will cost about 1,250,000 francs. The hospital employs 255 persons, of whom 35 are qualified physicians and surgeons; facilities are afforded for the clinical instruction of medical students, both men and women. The nursing arrangements are entrusted to a Protestant Order of Deaconesses, and in this important department of hospital work there is very great need for reorganisation and the introduction of a more thorough and practical method of training nurses, such as we enjoy in Great Britain. The need for this improvement is recognised by the medical and surgical staff, and the subject is, at the present time, receiving the careful consideration of the authorities.

According to the statistics included in the Report the average daily cost of each patient was 5.19 francs, which is equivalent to about 4s. 2d. of our money.

In view of the effects which the incidence of the National Insurance Act must inevitably have, the methods which have been adopted by the Swiss cantons are worthy of consideration. The general impression left upon my mind after visiting several public hospitals is that the patients are well cared for and very kindly treated, but there is great need for reform in nursing arrangements and for securing the general order and cleanliness which is so essential to the thorough efficiency of modern hospitals.

